# Diagnosis and therapeutic strategies for primary bladder mucinous adenocarcinoma: a case report and literature review

**DOI:** 10.3389/fonc.2025.1652375

**Published:** 2025-10-08

**Authors:** Xi Cheng, Weibo Wang, Lexing Yang, Supeng Tai, Junyue Tao, Yao Fu, Jun Zhou

**Affiliations:** ^1^ Department of Urology, The First Affiliated Hospital of Anhui Medical University, Hefei, Anhui, China; ^2^ Institute of Urology, Anhui Medical University, Hefei, Anhui, China; ^3^ Department of Pathology, The First Affiliated Hospital of Anhui Medical University, Hefei, Anhui, China; ^4^ Anhui Public Health Clinical Center, Hefei, Anhui, China

**Keywords:** primary bladder mucinous adenocarcinoma, signet-ring cell carcinoma, immunohistochemistry, chemotherapy, FOLFOX

## Abstract

Primary bladder mucinous adenocarcinoma (BMA) is an exceedingly rare and aggressive malignancy. We report a case of a 72-year-old male presenting with hematuria and dysuria. Imaging revealed a bladder mass, and histopathological examination following biopsy demonstrated characteristic extracellular mucin pools and signet-ring cells. Immunohistochemistry (IHC) was crucial for diagnosis, showing a negative profile for GATA binding protein 3 (GATA3) and Special AT-rich sequence-binding protein 2 (SATB2), along with membrane nuclear positivity for β-catenin. Endoscopic examination confirmed the absence of a primary gastrointestinal malignancy. The patient underwent robot-assisted laparoscopic radical cystoprostatectomy followed by three cycles of adjuvant chemotherapy with the FOLFOX regimen (5-fluorouracil, leucovorin, and oxaliplatin). No recurrence was observed during the 6-month follow-up. This case highlights the diagnostic challenges of BMA and emphasizes the importance of a multimodal diagnostic approach incorporating histopathology, immunohistochemistry, and endoscopy. The potential efficacy of adjuvant FOLFOX regimen is worth further exploration given the lack of standard therapeutic guidelines for this rare entity.

## Introduction

Mucinous adenocarcinoma predominantly arise in the gastrointestinal tract such as the stomach, appendix, or colon, or in the ovaries, while primary mucinous adenocarcinoma of the bladder is exceptionally rare, accounting for merely 2% of bladder malignancies. Distinguishing Primary bladder mucinous adenocarcinoma (BMA) from metastatic adenocarcinoma is often challenging due to overlapping histological features. Moreover, BMA is highly aggressive and often progresses rapidly. More than 50% of BMA cases are diagnosed at stage T3 or higher, and approximately 10% already show lymph node involvement or distant metastases at diagnosis. Therefore, early and accurate diagnosis is crucial. Herein, we present a representative BMA case to illustrate the value of multimodal diagnostic integration, including imaging, endoscopy, and Immunohistochemistry (IHC) evaluations. Concurrently, we review clinical outcomes of various therapeutic regimens reported in BMA management, aiming to enhance recognition of this rare aggressive entity and advance optimization of treatment strategies.

## Case report

A 72-year-old male presented to a local hospital with a 3-month history of dysuria and painless gross hematuria. Computed tomography (CT) revealed a heterodense lesion within the bladder cavity and left hydronephrosis. The patient underwent ureteroscopy with biopsy of the bladder lesion and bilateral ureteral stent placement to maintain upper urinary tract patency. The biopsy showed mucinous adenocarcinoma with focal signet-ring cell carcinoma (SRCC) features, raising the possibility of a metastatic adenocarcinoma. The patient was subsequently referred to our department for definitive diagnosis and treatment planning.

On admission, physical examination showed a soft abdomen without tenderness or palpable masses. The urine drained via the indwelling catheter was pale-red and contained blood clots. Digital rectal examination revealed a grade II enlarged prostate with firm consistency and no nodules.

Laboratory investigations, including complete blood count, hepatic and renal function panels, coagulation profile, and electrolyte levels, were unremarkable. Urinalysis demonstrated a red blood cell count of 2,849/μL and a white blood cell count of 531/μL. Serum tumor markers for gastrointestinal malignancy (carcinoembryonic antigen [CEA], alpha-fetoprotein [AFP], carbohydrate antigen [CA]125, CA19-9) and prostate-specific antigen (PSA) were within normal limits.

**Figure 1:**
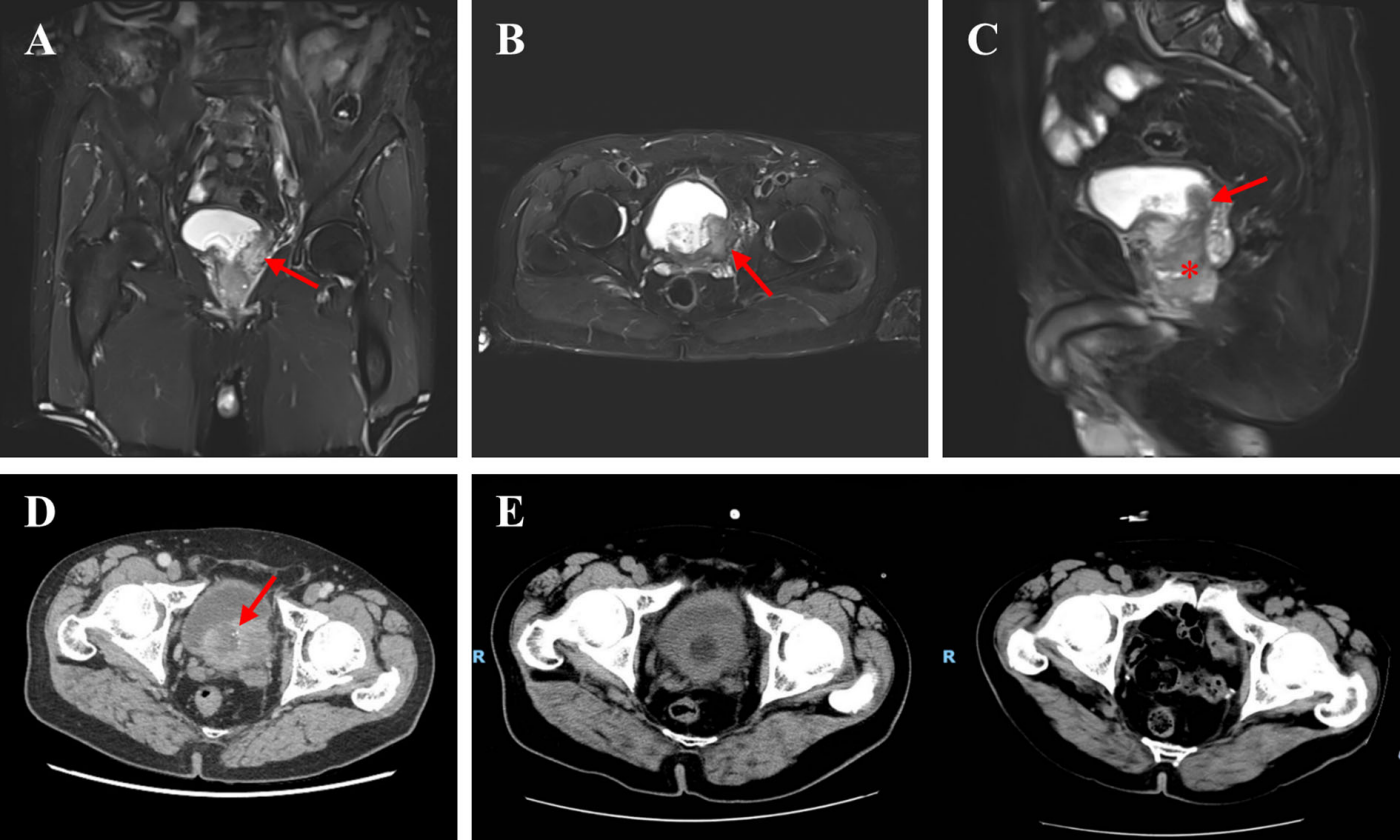
Preoperative and postoperative imaging of bladder mucinous adenocarcinoma. **(A–C)** T2-weighted pelvic MRI demonstrate an intravesical lesion (arrows) arising from the left posterior bladder wall in the coronal **(A)** axial **(B)** and sagittal **(C)** planes. The asterisk (*) in **(C)** indicates tumor invasion into the prostate. **(D)** CT urography reveals a posterior bladder wall mass with heterogeneous hypodensity (arrowheads), suggesting mucin deposition. **(E)** Postoperative CT at 6 months after radical cystectomy with pelvic lymphadenectomy demonstrates no pelvic fluid, bowel obstruction, or metastatic recurrence. CT, computed tomography; MRI, magnetic resonance imaging.

Contrast-enhanced magnetic resonance imaging (MRI) of bladder with diffusion-weighted imaging (DWI) identified an irregular focal thickening in the left posterior bladder wall, forming an intraluminal protrusion extending into the prostate (**Figures 1A–C**). Bilateral enlarged lymph nodes adjacent to the iliac vessels were detected. These findings were corroborated by contrast-enhanced CT urography, which additionally revealed irregular hypodense areas within the lesion, resembling mucin-filled cystic cavities (**Figure 1D**). To exclude gastrointestinal metastasis, combined esophagogastroduodenoscopy and colonoscopy were performed, demonstrating no evidence of primary gastrointestinal malignancy.

**Figure 2:**
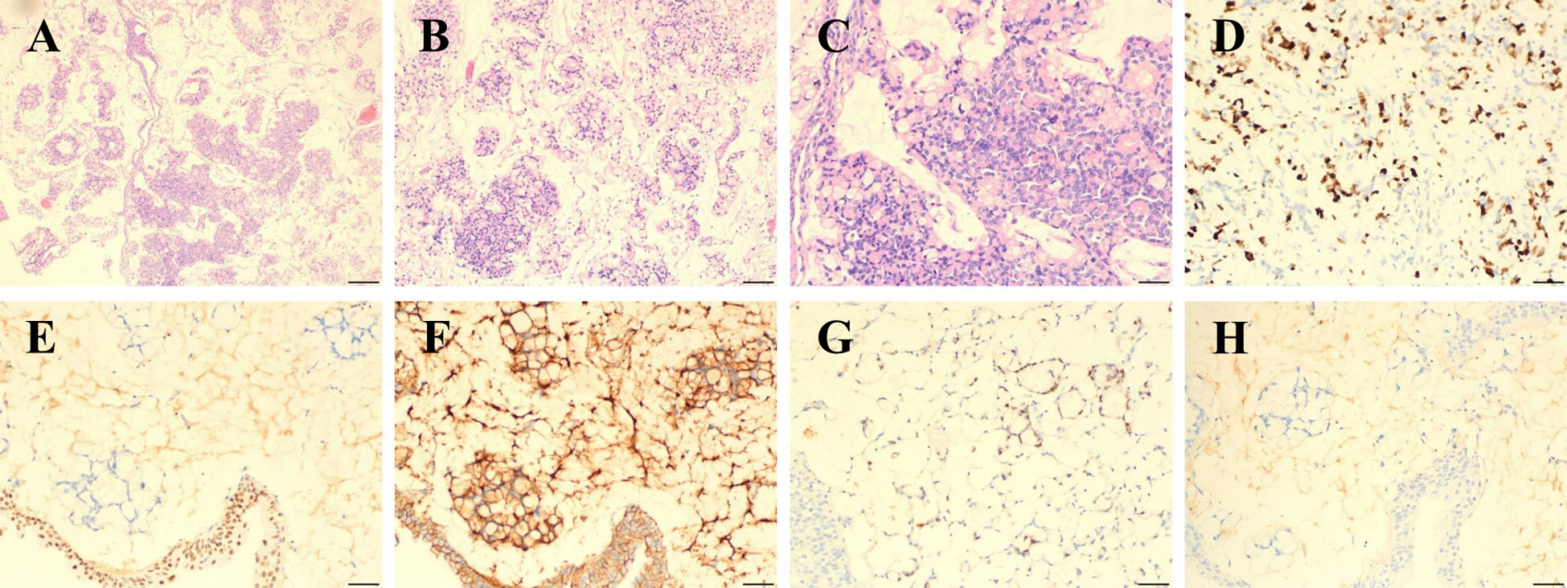
Histopathological and immunohistochemical features of primary bladder mucinous adenocarcinoma. **(A, B)** H&E staining (×40) shows extracellular mucin pools with floating tumor cell clusters. **(C)** High-power view (×200) reveals signet-ring cells (arrows) exhibiting cytoplasmic mucin accumulation and crescent-shaped nuclei. **(D–H)** IHC profile: **(D)** Ki-67 (+, ~57% proliferation index); **(E)** GATA3 (−); **(F)** β-catenin (membranous +); **(G)** CDX2(+); **(H)** SATB2 (−).H&E staining, Hematoxylin-eosin staining, IHC, Immunohistochemistry; GATA3, GATA binding protein 3; CDX2, caudal type homeobox 2; SATB2, Special AT-rich sequence-binding protein 2.

Based on these findings, we performed additional IHC staining on the biopsy tissue sections obtained from the external hospital (**Figure 2**). IHC revealed negative expression of cytokeratin 7 (CK7), cytokeratin 20 (CK20), GATA binding protein 3 (GATA3), Special AT-rich sequence-binding protein 2 (SATB2), and Villin, with scattered positivity for caudal type homeobox 2 (CDX2). β-catenin showed membrane positivity without nuclear staining, and the Ki-67 proliferation index was 60%. These collective features strongly suggested the possibility of primary bladder adenocarcinoma (PBA).

The patient subsequently underwent Da Vinci robot-assisted laparoscopic radical cystoprostatectomy with pelvic lymph node dissection, appendectomy, and urinary diversion via Wallace ureteroileal anastomosis. Intraoperative frozen sections of both ureteral margins were negative for tumor involvement.

Postoperative histological examination revealed tumor cells with glandular differentiation invading the full thickness of the bladder wall and involving the prostate. Abundant extracellular mucin formed extensive mucinous lakes, with some cells demonstrating intracellular mucin accumulation and signet-ring morphology. Metastatic adenocarcinoma was identified in bilateral pelvic lymph nodes. The final pathological diagnosis confirmed advanced primary mucinous adenocarcinoma of the bladder, staged as pT4N2Mx.

**Figure 3:**
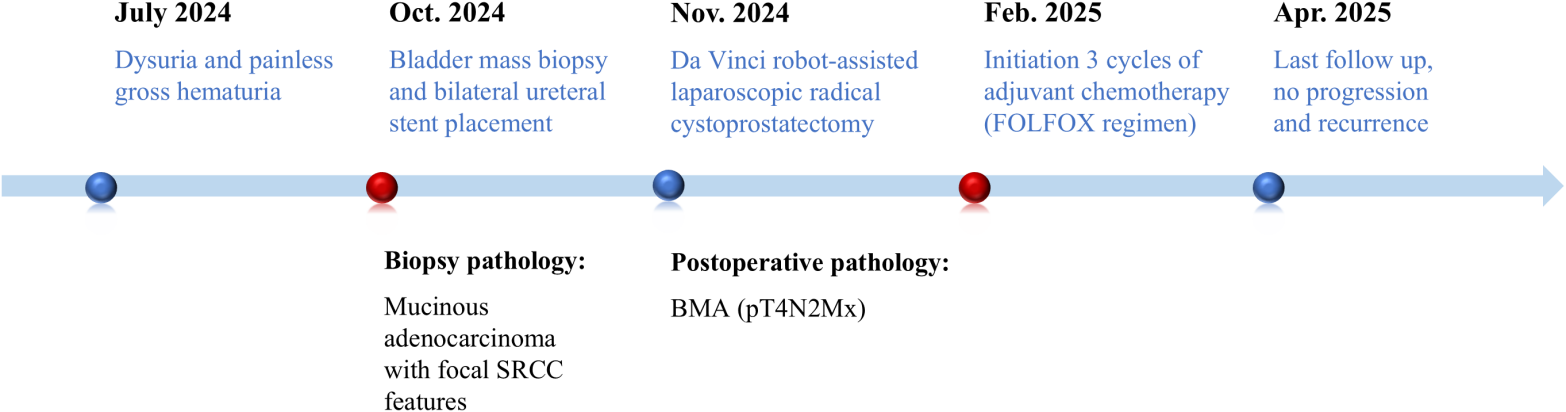
Timeline of the treatment. SRCC, signet-ring cell carcinoma.

The operation was successful, and the patient subsequently received 3 cycles of the FOLFOX regimen. Grade 2 myelosuppression occurred during treatment cycles, which was successfully managed with granulocyte colony-stimulating factor (G-CSF) support. At the 6-month follow-up, the patient remained clinically stable, with no signs of recurrence on abdominopelvic CT (**Figure 1E**). Clinical evaluations every 3 months and annual CT scans were recommended for surveillance. The key events and corresponding timelines in this case are summarized in **Figure 3**.

## Discussion

PBA is a rare malignancy, accounting for only 0.5-2% of all bladder malignancies (1). The peak incidence occurs in the sixth decade of life, with a male predominance (2). The pathogenesis of PBA is predominantly linked to chronic irritation, such as urinary retention, bladder calculi, or chronic cystitis. This persistent insult triggers a metaplastic process in the bladder mucosa, which evolves through a series of stages including urothelial hyperplasia, cystitis glandularis, and cystitis cystica, eventually culminating in the development of adenocarcinoma (3, 4).

PBA encompasses several histological subtypes, including signet-ring cell, clear cell, enteric, hepatoid, mucinous, and adenocarcinoma not otherwise specified (NOS) (5, 6). BMA, a relatively rare and highly aggressive subtype, accounts for 15% of all primary bladder adenocarcinomas (7). Histologically, BMA is characterized by tumor cells arranged in nests floating within extensive extracellular mucin pools. In some cases, tumor cells contain intracellular mucin, which displaces the nucleus and cytoplasm to the periphery, resulting in a signet-ring cell appearance (8). Consequently, BMA is frequently observed to harbor a signet-ring cell component (9). When signet-ring cells predominate within a mucinous background, the tumor is classified as a signet-ring cell mucinous adenocarcinoma (SRCMA) (2, 10).

As a non-urachal origin bladder adenocarcinoma, BMA predominantly arises in the posterior wall and trigone of the bladder rather than the dome (11). Although a minority of patients exhibit pathognomonic mucus in urine, the vast majority of BMA cases present with nonspecific symptoms analogous to conventional urothelial carcinoma (UC), including hematuria, urinary frequency, dysuria, and pelvic pain (9). The lack of specific symptoms often leads to a delayed diagnosis. Approximately 50% of BMA patients are diagnosed at stage T3 or T4. Moreover, nearly 10% present with lymph node or distant metastases at initial diagnosis (12).

In terms of supporting examinations, BMA typically demonstrates no abnormalities in serologic tumor markers (13). While CT and MRI scans can help understand the tumor size, anatomical location, depth of invasion, and metastatic spread, they exhibit limited specificity in differentiating BMA from UC. Therefore, cystoscopy with histopathological biopsy is necessary for establishing a definitive diagnosis. Furthermore, it is essential to distinguish BMA from other adenocarcinomas that secondarily involve the bladder, either through metastasis or direct invasion, such as colorectal, prostatic, or endometrial adenocarcinomas. Esophagogastroduodenoscopy and colonoscopy can help to exclude potential primary malignancies in the gastrointestinal tract. In this case, a comprehensive IHC analysis was critical. It ruled out metastatic adenocarcinoma and confirmed the primary bladder adenocarcinoma diagnosis (14). Urothelial-specific markers such as GATA3, p63, and uroplakin II/III are typically negative in BMA. β-catenin usually shows nuclear positivity in colorectal adenocarcinoma but demonstrates membranous and cytoplasmic staining in primary bladder adenocarcinoma, serving as an important diagnostic marker (9, 15). Classic colorectal adenocarcinomas are typically positive for CDX2, SATB2, and villin, whereas BMA often exhibits negative or weakly positive expression of these markers (16–18). Notably, recent studies have indicated that CK7 and CK20 immunostaining lacks specificity in distinguishing primary from secondary adenocarcinomas, as 29% of primary bladder adenocarcinomas exhibit an IHC profile overlapping with colorectal adenocarcinoma, characterized by CK7 negativity and CK20 positivity (19). As a novel diagnostic approach, molecular testing remains under investigation for its potential role in diagnosing BMA and guiding therapeutic strategies. Pires-Luis et al. performed next-generation sequencing and identified *KRAS*, *GRIN2A*, and *AURKB* as the most frequent genetic alterations in BMA (20).

Regarding therapeutic management, no consensus exists on clinical strategies for BMA due to its rarity, aggressive behavior, and frequent delayed diagnosis. Radical cystectomy with pelvic lymphadenectomy is generally considered the primary treatment option for surgically resectable BMA (21). However, a retrospective analysis of 426 BMA patients revealed no survival advantage for muscle-invasive cases treated with radical surgery versus partial or local resection. This finding indicates that bladder-preserving approaches may be feasible (22).

According to the US National Comprehensive Cancer Network (NCCN) guidelines, postoperative adjuvant chemoradiotherapy may be considered for advanced or unresectable bladder adenocarcinoma (23). However, due to limited clinical trial evidence, no consensus exists regarding the optimal chemotherapy regimens or their efficacy. Initially, chemotherapy protocols commonly used for UC have been empirically applied to BMA (**Table 1**). Wajpeyi et al. and Di Maida et al. reported two cases of BMA with signet-ring cell components. In both cases, adjuvant chemotherapy with gemcitabine and paclitaxel was administered after radical cystectomy but yielded suboptimal outcomes. Both patients developed inguinal lymph node recurrence within 8 and 10 months postoperatively, respectively, and succumbed to the disease shortly thereafter (10, 24). In contrast, Ball et al. described a successful case treated with carboplatin, gemcitabine, and paclitaxel, where the patient remained recurrence- and metastasis-free for 90 months after radical cystectomy and adjuvant chemotherapy (25). Additionally, the gemcitabine plus cisplatin (GC) regimen has been sporadically reported in BMA management (26–28). Subsequently, given the adenocarcinoma characteristics of BMA, chemotherapy regimens typically used for gastrointestinal adenocarcinomas have also been implemented, achieving sporadically successful outcomes (**Table 2**). Notably, given the established efficacy of FOLFOX in gastrointestinal-derived mucinous adenocarcinomas, some researchers have explored its application in BMA. Tatil et al. reported a case of a BMA patient who experienced disease progression after four cycles of the GC regimen; subsequent switch to FOLFOX resulted in a 10-month clinical remission, suggesting potential efficacy in this setting (29). Another patient diagnosed with SRCC accompanied by multiple pulmonary metastases achieved a complete response following FOLFOX chemotherapy (30). In recent cases, the FOLFOX regimen has increasingly been adopted as a first-line option (9, 13). Similarly, combinations of cisplatin with 5-fluorouracil (5-FU) or tegafur-gimeracil-oteracil (S-1), which are commonly used in gastrointestinal adenocarcinomas, have also demonstrated efficacy in BMA management (31, 32).

**Table 1:** Reported outcomes of urothelial carcinoma-based chemotherapy regimens in bladder mucinous adenocarcinoma.

Investigator (Year)	Pathological type	Age/Sex	Stage	Treatment modality	Drugs	Cycles	Treatment response/status	Survival outcome (Months)	Ref.
Wajpeyi et al.(2023)	MA+SRC	60/M	T4aN0Mx	Surgery + Adjuvant Chemo	Gemcitabine and Taxol	2	Recurrence (lymph node, 8 month)	OS 19, DFS 8 (Died)	(10)
Di Maida et al.(2016)	MA+SRC	57/M	T4aN1M0	Surgery + Adjuvant Chemo	Gemcitabine and Taxol	2	Recurrence (lymph node, 10 month)	OS 19, DFS 8 (Died)	(24)
Ball et al.(2016)	MA	33/M	T4aN0Mx	Surgery + Adjuvant Chemoradiation	Carboplatin, Taxol and Gemcitabine	2	NED	OS 96, DFS 90 (Alive)	(25)
Cobo-Dols et al.(2006)	SRCC*	53/M	T4aN0M0	Surgery + Adjuvant Chemo	GC regimen	4	NED	OS 8, DFS 8 (Alive)	(26)
Sanish et al.(2011)	SRCC	48/M	T3N0M0	Surgery + Adjuvant Chemo	GC regimen	4	NED	OS 12, DFS 12 (Alive)	(27)
Lendorf et al.(2018)	SRCC	62/M	T4NxM1	Chemotherapy only	GC regimen	3	PD	OS 4(Died)	(28)
Ota et al. (1995)	SRCC	54/M	T2N0M0	Neoadjuvant Chemoradiation + Surgery	MTX and Cisplatin	4	NED	OS 22, DFS 22 (Alive)	(36)

*The SRCC cases included in this table all exhibited extracellular mucin lakes. Although the proportion of signet-ring cells influences subtype classification, their shared mucin-secreting phenotype and treatment response patterns support combined analysis. MA, mucinous adenocarcinoma; SRCC, signet ring cell carcinoma; MA+SRC, mucinous adenocarcinoma with signet-ring cells; GC regimen, gemcitabine and cisplatin; MTX, methotrexate; CR, complete response; PR, partial response; SD, stable disease; PD, progressive disease; NED, no evidence of disease.

**Table 2:** Therapeutic outcomes of gastrointestinal adenocarcinoma-inspired regimens.

Investigator (Year)	No. of cases	Pathological type	Age/ Sex	Stage	Treatment modality	Drugs	Cycles	Treatment response/status	Survival outcome (Months)	Ref.
Wang et al.(2022)	1	MA+SRC	62/F	T3NxM1	Surgery + Adjuvant Chemo	FOLFOX	2	SD	OS 2(Alive)	(13)
Tatli et al. (2012)	1	MA	65/M	T4N0M0	Surgery + Adjuvant Chemo	FOLFOX	2	NED	OS 20, DFS 20 (Alive)	(29)
Tatli et al. (2015)	1	SRCC*	41/M	T4N1M1	Surgery + Adjuvant Chemo	FOLFOX	12	CR	OS 12, DFS 12 (Alive)	(30)
Hamakawa et al.(2013)	1	SRCC	53/M	T3bN0M0	Surgery + Adjuvant Chemo	S-1 and cisplatin	3	NED	OS 90, DFS 90 (Alive)	(31)
Romics et al.(2008)	1	SRCC	45/F	T3bN0M0	Surgery + Adjuvant Chemo	cisplatin and 5-FU	4	NED	OS 60, DFS 60 (Alive)	(32)
Messina et al.(2015)	1	MA	64/M	T4bN0Mx	Surgery + Adjuvant Chemo	Capecitabine	3	Recurrence (distant, 10 month)	OS 9, DFS 6 (Alive)	(35)
Logothetis et al.(1985)	5	MA/ MA+SRC	Median 60/M	T3-4Nx M0-1	Chemotherapy only	Doxorubicin, mitomycin-C, and 5-FU	22or18	CR 20%, PR40%, PD 20%	Average OS 11.6	(37)

*The SRCC cases included in this table all exhibited extracellular mucin lakes. Although the proportion of signet-ring cells influences subtype classification, their shared mucin-secreting phenotype and treatment response patterns support combined analysis. MA, mucinous adenocarcinoma; SRCC, signet ring cell carcinoma; MA+SRC, mucinous adenocarcinoma with signet-ring cells; GC regimen, gemcitabine and cisplatin; MTX, methotrexate; CR, complete response; PR, partial response; SD, stable disease; PD, progressive disease; NED, no evidence of disease.

The efficacy of postoperative radiotherapy in BMA remains poorly documented. However, a retrospective study from Egypt demonstrated that adjuvant radiotherapy following cystectomy provided favorable survival rates and local control for PBA patients, though with limited efficacy in controlling distant metastases, particularly in BMA and SRCC (33).

Due to frequent delays in detection and diagnosis, BMA is generally associated with a poor prognosis. Retrospective studies indicate a 5-year disease-free survival (DFS) rate of 36% for BMA and 7% for SRCC, both significantly lower than those of UC or other adenocarcinoma subtypes (33). Song et al. analyzed 426 BMA patients from the SEER database, reporting a median overall survival (OS) of 47 months (22). Notably, this analysis revealed no significant survival difference between muscle-invasive and non-muscle-invasive BMA patients, suggesting limited prognostic value of T-stage classification in BMA. It has been reported that postoperative elevation of CEA levels may assist in assessing SRCC malignancy and monitoring disease progression (34), with similar patterns observed in BMA cases (35). However, whether these CEA level changes are associated with distant metastasis remains undetermined.

In summary, the diagnosis and management of BMA remain challenging. Due to its rarity, current understanding is largely based on isolated case reports, which lack long-term follow-up data and dedicated clinical studies. Therefore, future efforts should focus on establishing multi-institutional registries and promoting collaborative research to improve diagnostic and treatment strategies.

## Data Availability

The original contributions presented in the study are included in the article/supplementary material. Further inquiries can be directed to the corresponding authors.

## Glossary

BMA: primary bladder mucinous adenocarcinoma
IHC: immunohistochemistry
CT: computed tomography
SRCC: signet-ring cell carcinoma
CEA: carcinoembryonic antigen
AFP: alpha-fetoprotein
CA125: carbohydrate antigen 125
CA19-9: carbohydrate antigen 19-9
PSA: prostate-specific antigen
MRI: magnetic resonance imaging
DWI: diffusion-weighted imaging
CK7: cytokeratin 7
CK20: cytokeratin 20
SATB2: special AT-rich sequence-binding protein 2
GATA3: GATA binding protein 3
CDX2: caudal type homeobox 2
PBA: primary bladder adenocarcinoma
G-CSF: granulocyte colony-stimulating factor
NOS: not otherwise specified
SRCMA: signet-ring cell mucinous adenocarcinoma
p63: tumor protein p63
UC: urothelial carcinoma
NCCN: National Comprehensive Cancer Network
5-FU: 5-fluorouracil
S-1: tegafur-gimeracil-oteracil
NCCN: US National Comprehensive Cancer Network
DFS: disease-free survival
OS: overall survival.
